# The role of plain radiography in paediatric wrist trauma

**DOI:** 10.1007/s13244-012-0181-0

**Published:** 2012-06-26

**Authors:** Annelie Slaar, Abdelali Bentohami, Jasper Kessels, Taco S. Bijlsma, Bart A. van Dijkman, Mario Maas, Jim C. H. Wilde, J. Carel Goslings, Niels W. L. Schep

**Affiliations:** 1 Department of Radiology, Academic Medical Centre, University of Amsterdam, P.O. Box 22660, 1100 DD Amsterdam, The Netherlands; 2 Trauma Unit, Department of Surgery, Academic Medical Centre, University of Amsterdam, P.O. Box 22660, 1100 DD Amsterdam, The Netherlands; 3 Department of Surgery-Traumatology, Spaarne Hospital, Spaarnepoort 1, 2134 TM Hoofddorp, The Netherlands; 4 Department of Surgery-Traumatology, Flevo Hospital, Hospitaalweg 1, 1315 RA Almere, The Netherlands; 5 Department of Paediatric Surgery, Academic Medical Centre, University of Amsterdam, P.O. Box 22660, 1100 DD Amsterdam, The Netherlands; 6 Trauma Unit, Department of Surgery, Academic Medical Center, Meibergdreef 9, 1105 AZ Amsterdam, The Netherlands

**Keywords:** Wrist injuries, Paediatrics, Radiography, Fractures, Bones, Epidemiology

## Abstract

**Objectives:**

Acute wrist trauma in children is one of the most frequent reasons for visiting the emergency department (ED). Radiographic imaging in children with wrist trauma is mostly performed routinely to confirm or rule out a fracture. The aim of this study was to determine how many radiographs of the wrist show a fracture in children following wrist trauma.

**Methods:**

A retrospective cohort study was performed in three Dutch hospitals from 2009–2010. Data were extracted from patient records and radiographic reports.

**Results:**

Of the 1,223 children who presented at the ED after a wrist trauma, 51 % had a wrist fracture. The peak incidence of having a wrist fracture was at the age of 10 years; 65 % of the children younger than 10 years of age had a wrist fracture. Of all the patients without a wrist fracture, 74 % were older than 10 years of age.

**Conclusion:**

Almost half of the paediatric patients with a trauma of the wrist had normal radiographs. The development of a clinical decision rule to determine when a radiograph of the wrist is indicated following acute wrist trauma is needed. This could likely reduce the number of radiographs.

***Main Messages*:**

- *Fifty-one percent of the children with wrist trauma have a wrist fracture*.
- *Peak incidence of having a wrist fracture is at the age of 10 years*.
- *Sixty-five percent of the children younger than 10 years of age had a wrist fracture*.
- *Of all the patients without a wrist fracture, 74 % were older than 10 years of age*.
- *The development of a clinical decision rule to reduce the number of radiographs is needed*.

## Introduction

Fractures in children (0–16 years of age) account for 10–25 % of all paediatric injuries [1, 2]. In studies performed from 1950–2007, the percentage of distal forearm or wrist fractures in children varied from 20–36 % of all the paediatric fractures [2–9]. The most common location was the distal forearm, while falling is the main trauma mechanism [3]. Over the last decade a 13 % increase in the incidence of children with a fracture was observed [3].

Due to this apparent rise in incidence, health care costs are increasing; in the USA, the estimated costs per year for childhood forearm fractures already exceed 2 billion dollars [10].

In most hospitals radiographic imaging in children with wrist trauma is performed routinely to confirm or rule out a fracture. Because of this routine, unnecessary costs are incurred and emergency department (ED) waiting time is extended.

The aim of this study was to determine how many radiographs of the wrist showed a fracture in children presenting to the emergency department following wrist trauma.

## Patients and methods

### Patients and study outline

A retrospective cohort study was performed in three Dutch hospitals from 2009–2010. The participating hospitals included one university, one non-academic teaching and one non-teaching hospital. All consecutive children, 3–16 years of age, for whom a wrist radiograph had been performed after wrist trauma were included.

The wrist was defined as: the carpal bones and the distal third of the forearm (i.e., ulna and radius).

A fracture was defined as a disruption of at least one of the cortices of the wrist or an osseous avulsion at the site of attachment of a ligament or tendon. Bilateral fractures were recorded as two fractures.

In all patients a posterior-anterior and lateral radiograph of the wrist was performed. In patients suspected of having a scaphoid fracture, additional radiography of the scaphoid was performed.

Exclusion criteria included patients who had suffered wrist injury more than 72 h before presentation and patients who were referred with radiographs from another hospital or had returned for reassessment of the same injury. Multitrauma patients (Injury Severity Score ≥16) were also excluded.

The following demographic and clinical data were scored: sex, age, fracture type, affected side and type of hospital.

### Statistics

All analyses were performed using PASW statistics, version 18.0. (SPSS, Chicago, IL, USA). Normality of continuous data was tested with the Shapiro-Wilk and Kolmogorov-Smirnov test, and by inspecting the frequency distributions (histograms). The homogeneity of variances was tested using Levene’s test. Continuous variables are expressed as the mean ± standard deviation (SD) if normally distributed, or otherwise as the median and interquartile range (IQR). Categorical variables are expressed as frequency (percentage).

Univariate analysis was performed to test the difference in the primary and secondary outcome measures between patients with a distal radius fracture and patients without. Continuous data were tested using Student’s t-test (parametric data) or a Mann-Whitney U-test (non-parametric data). Chi-square analysis was used for statistical testing of categorical data. In all tests, a p-value of less than 0.05 was considered to indicate statistical significance.

## Results

In total, 1,223 patients were included in the study. Patient demographics are shown in Table 1. More males were diagnosed with a wrist fracture, (*P* < 0.001). There was a significant preponderance of left-side fractures (*P* < 0.03). The difference in fracture percentages among the three hospitals was not significant (*P* = 0.5).

**Table 1:** Characteristics of patients with or without wrist fracture

Variable	Fracture (%)	No fracture	Total group
Mean age in years (SD)	10 (3.2)	12	11 (3.1)
Sex			
Boys	356 (58)	253	609
Girls	271 (44)	343	614
Hospital			
Non-teaching hospital	224 (54)	193	417
University (teaching) hospital	154 (50)	154	308
Teaching hospital	249 (50)	249	498
Injured side			
Left	367 (54)	312	679
Right	260 (4.8)	284	544

In total, 627 (51 %) children were radiologically diagnosed with a wrist fracture. In 596 patients (49 %), the radiograph showed no wrist fractures.

**Fig. 1:**
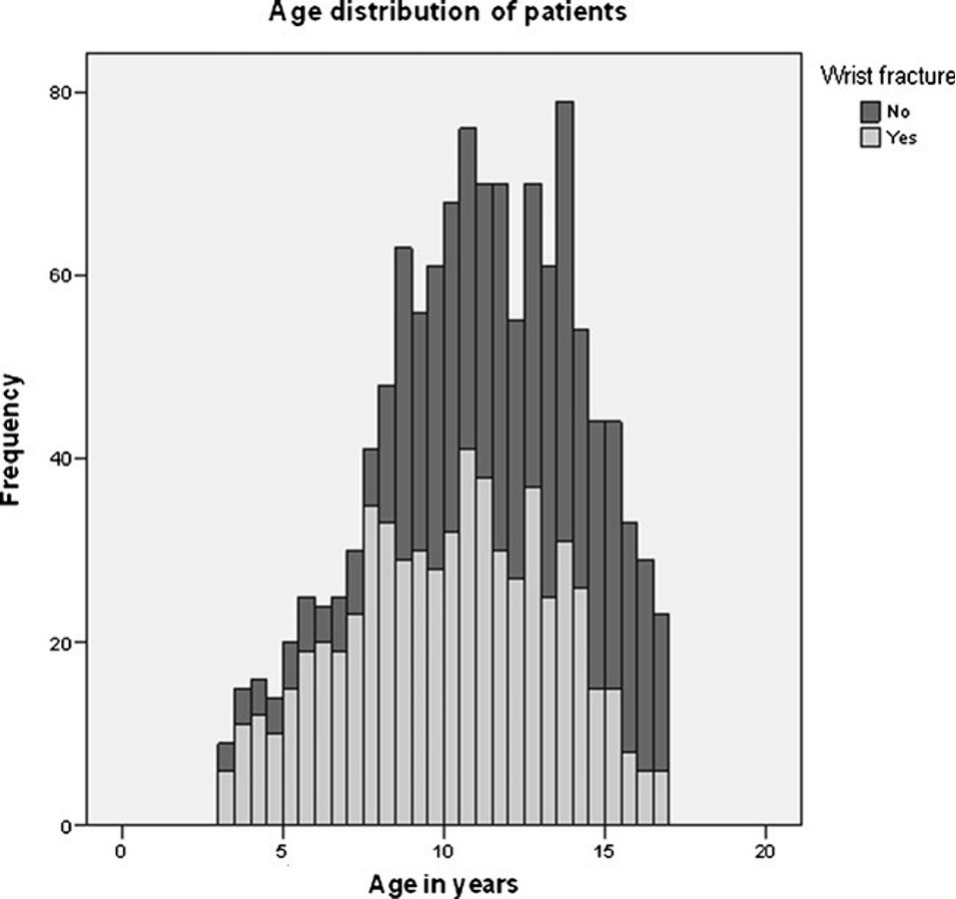
Age distribution and incidence of wrist fractures

The age distribution and the incidence of wrist fractures is shown in Fig. 1.

Table 2 shows the number of patients with a wrist fracture stratified according to age older and younger than 10 years. According to the age distributionl we used a cutoff value of the age of 10. Sixty-five percent of the children younger than 10 years of age had a wrist fracture; for children older than 10 years of age, this was 43 %. Of all the patients without a wrist fracture, 74 % were older than 10 years of age.

**Table 2:** Distribution of age according to fracture, yes/no

Age	Yes (%)	No (%)	Total
0 = 10	290 (65 %)	157	447
10	337	439 (74 %)**	776
Total	627	596	1,223

P-value: <0.001

*Percentage of patients under the age of 10 with a wrist fracture

**Percentage of patients without a wrist fracture over the age of 10

The most frequently diagnosed fracture type was a distal radius fracture, with a cumulative percentage of 73 %. No bending or pathological fractures were seen amongst all the patients.

## Discussion

Our study shows that in 51 % of the children for whom plain radiography of the wrist was performed following wrist trauma, a fracture of the wrist was diagnosed. In other words, in half of the patients no fracture was diagnosed on the radiograph.

These negative series can be qualified as potentially unnecessary. Therefore, based on these findings, it can be concluded that the current use of radiography in patients with acute wrist trauma is suboptimal.

Petit et al. tested the effectiveness of radiographs in children and found that 43 % of the children with wrist trauma showed no fracture [11]. However, this study was performed over a period of only 24 weeks with a relative small sample size (n = 327) in patients with wrist trauma.

We assume that our percentages are higher because of the more articulate parents who demand a wrist radiograph and because of the more defensive way of practicing medicine nowadays.

Our study shows that in patients younger than 10 years of age, the a priori chance of having a wrist fracture was 65 %. Of all the patients without a wrist fracture, 74 % were older than 10 years of age. We think this difference is based on the fact that younger children tend to inactivate their arm after serious trauma, so parents are more aware of the severity of the trauma. Also younger children continue playing if the injury is not severe. In older children, such differentiation is hard for parents to make. Also secondary gain, such as getting attention and not having to participate in school/sports, might play a role in older more than in younger children.

An important limitation of our study is that children who did not undergo radiographic examination were not included in the study. Therefore, it is not known what the total population was of patients presenting to the emergency department after wrist trauma. This could have biased the selection of our study population, because it might be that some of those patients had a fracture, and those patients were not included.

Another limitation of our study was the lack of follow-up data. Because of this, we do not know our false-negative rate. We know from the hospital database that none of the children came back to our hospital with a missed fracture.

Swischuk et al. publiced that subtle fractures in children can be easily missed if no comparative views are considered; because we performed no follow-up on our patients, the number of patients in whom we missed subtle fractures is not known [12].

The overall costs declared by Dutch insurance companies for plain radiography of the wrist in our country is approximately 48 euros (€). Thus, a total of approximately € 28,608/year is spent on radiographs that show no osseous injury in those three hospitals. Not all of those radiographs are useless because they are helpful to rule out a fracture. However, only a small reduction in the number of radiographs may already result in considerable health-care cost reductions.

Besides the money-saving aspect, by reducing the number of radiographs, also the reduction of radiation dose is important. The mean estimated radiation dose of a radiograph of the wrist in children is less than 0.06 μSv [13]. Although the radiation dose of a radiograph of the wrist is low, it is important to reduce the radiation dose. For this reason, comparative views are limited in our experience. According to the ALARA principles, we should expose patients to as little radiation as possible [14].

Stiell et al. conducted a study that showed that the vast majority of ankle traumas in adult patients was radiographically examined upon presentation [15]. They established that only 9.3 % of these patients were found to have significant malleolar fractures and subsequently developed the renowned *Ottowa Ankle Rules*. After implementation of this clinical decision rule, a relative decrease (RD) of 28 % of ankle radiography was recorded in the intervention hospital, whilst the control site showed a 2 % increase. A reduction of time spent in the emergency department (116 min vs. 80 min) was also found without missed fractures or patient discontent [16].

A reduction in the number of radiographs can potentially be achieved if children with wrist trauma are not routinely referred for radiological examination. This is the reason why we are developing a clinical decision rule in children with wrist trauma to form a potential solution to this problem [17]. The clinical decision rule we are developing is in analogy to the Ottawa Ankle Rules. The clinical physician has to perform a regular clinical examination. If a patient fulfils the criteria of the clinical decision rule, a radiograph of the wrist will be made. Patients who do not fulfil those criteria will not undergo radiographic imaging. Because understanding and participation during clinical examination are necessary, children younger than 3 years of age are excluded, like in this study. The main goal of this decision rule is to reduce the amount of unnecessary imaging, taking into account that the number of missed fractures has to be as small as possible.

## Conclusion

Almost half of the paediatric patients with trauma of the wrist had normal plain radiographs. Therefore, the number of patients who are sent for radiographic imaging can be reduced, potentially without hampering clinical care.

Of all the patients without a wrist fracture, 74 % were older than 10 years of age.

The development of a clinical decision rule to determine when a radiograph of the wrist is indicated following acute wrist trauma is needed. This could likely reduce the number of radiographs.
